# The art of being mentally healthy: a study to quantify the relationship between recreational arts engagement and mental well-being in the general population

**DOI:** 10.1186/s12889-015-2672-7

**Published:** 2016-01-05

**Authors:** Christina Davies, Matthew Knuiman, Michael Rosenberg

**Affiliations:** 1 School of Population Health (M431), The University of Western Australia, 35 Stirling Hwy, Crawley, 6009 Western Australia Australia; 2 School of Sport Science, Exercise & Health, The University of Western Australia, 35 Stirling Hwy, Crawley, 6009 Western Australia Australia

**Keywords:** Arts, Culture, Mental health, Mental well-being, Population health, Health promotion, Warwick-Edinburgh Mental Well-being Scale

## Abstract

**Background:**

Little is known about the dose–response relationship between recreational arts engagement (for enjoyment, entertainment or as a hobby, rather than therapy) and mental well-being in the general population. The quantification of this relationship is of value to: (1) health professionals, clinicians and researchers interested in utilising the arts as a method for improving mental health; (2) to health promoters and policy makers in the development of population based health messages, policy and practice; and (3) to members of the general public in maintaining or improving their own well-being. As guided by theories of social epidemiology and the biopsychosocial model of health, the first aim of this study was to determine if there was a relationship between arts engagement (hours per year) and mental well-being in the general population. If an association was demonstrated, the second aim was to quantify this relationship.

**Methods:**

A random sample of 702 Western Australian adults aged 18+ years (response rate = 71 %) were invited to take part in a telephone survey. The survey took 15 min to complete and included questions about arts engagement, mental well-being, demographics and potential confounders/effect modifiers. The dependent variable was subjective mental well-being (Warwick-Edinburgh Mental Well-being Scale, WEMWBS). The independent variable was hours engaged in the arts in the last 12 months.

**Results:**

Respondent engagement in the arts ranged from zero to 1572 hours/year (mean = 100.8 hours/year, SD = 206.0). The prevalence of engagement was 83 %. The average WEMWBS score was 53 (SD = 7.4). After adjustment for demographics (i.e. sex, age group, location, income, education, marital status, children), general health, sports engagement, religious activities and holidays, respondents with high levels of arts engagement (100 or more hours/year, WEMWBS score = 55) had significantly better mental well-being than those with none (0 hours/year, WEMWBS score = 53), low (0.1–22.9 hours/year, WEMWBS score = 52) and medium (23–99.9 hours/year, WEMWBS score = 53) levels of engagement (*p* = 0.003). Respondents with none, low and medium arts engagement had similar WEMWBS scores (*p* = 0.358). The relationship between arts engagement and WEMWBS was nonlinear with evidence of a minimum threshold at 100 or more hours/year (*p* = 0.0006).

**Conclusion:**

Evidence of an arts-mental health relationship was found in this study. Those who engaged in 100 or more hours/year of arts engagement (i.e. two or more hours/week) reported significantly better mental well-being than other levels of engagement. The suitability of the arts as a population based strategy to influence the mental well-being of the general population should be investigated further.

## Background

With an emphasis on self-expression, creativity, enjoyment and social inclusion the arts are receiving increasing attention from health professionals, researchers, clinicians, policy makers and the general community as a means of improving population health and mental well-being [1–3]. Evidence of the benefits of recreational arts engagement has been mounting since the 1990s [4], yet little is known about how much arts engagement is needed for good mental health (i.e. dose–response). Good mental health or being ‘mentally healthy’ [5] can be defined as a state of well-being whereby an individual is able to contribute to their community, cope with the stresses of everyday life, is able to realise their potential and work productively [6]. Good mental health is essential for individual and community well-being. Poor mental health is a leading cause of mortality, disability and burden of disease [7]. World-wide, over 450 million people suffer from mental illness [8]. Overall, women compared to men; unmarried compared to married/de-facto; single parent with children compared to couple only households and younger compared to older people experience higher rates of mental illness [9, 10]. Engagement in physical activity [11, 12], spirituality [13], good general health [5], and holidays [14] are associated with mental well-being, while poor education and low income are associated with a higher prevalence of mental disorders [9, 15].

### Arts engagement

Arts engagement can be defined as active (e.g. making art) or receptive (e.g. attending concerts) involvement in creative events or activities within a variety of art forms such as the performing arts, visual arts and literature [16]. Arts engagement by members of the general population is high. For example, it is estimated that 78 % of British (16+ years), 86 % of Australian (15+ years), 85 % of New Zealander (15+ years), and 99 % of Canadian adults (15+ years) take part in creative events and activities each year [17–20].

### Arts engagement and mental health

Clinical studies have found that arts engagement promotes patient recovery, relaxation and reduces patient stress, anxiety and depression [21–23]. In cancer patients the arts have been used to enhance quality of life after medical treatment [24]. Arts programs for people experiencing mental ill-health have been linked with improved confidence, self-esteem and self-understanding [25, 26]. In the elderly, arts engagement reduces depressed mood, enhances self-worth and promotes positive aging [27, 28]. Arts programs for young people have been linked with improved motivation, self-image, hope for the future and self-esteem [29]. Where population-based studies have been conducted, arts engagement has been associated with perceptions of happiness and stress reduction [30, 31].

### Study aims

The arts may have a unique contribution to make to population health however, evidence-based, dose–response research about (1) the general population, and (2) the art people do as part of their everyday life, for enjoyment, entertainment or as a hobby, rather than therapy, is limited [32–34]. As pressure on health resources grow, many are calling for holistic solutions that provide members of the general population with knowledge, choice and the capacity to attain higher levels of wellness and self-care [35, 36]. A better understanding of the arts-mental health relationship could contribute to population-based health messages, strategies, policy and practice (e.g. social prescribing or arts-on-prescription programs) [37]. As guided by theories of social epidemiology [38] and the biopsychosocial model of health [39], the first aim of this study was to determine if there was a relationship between arts engagement and mental well-being in the general population. If an association was demonstrated, the second aim was to quantify this relationship.

## Methods

### Participant recruitment and survey

Between September 2011 and May 2012, telephone interviews were conducted with the Western Australian community (18+ years). Western Australia is representative of the broader Australian population in terms of key health and socio-demographic indicators [40]. Residential telephone numbers were randomly selected from the Australian Electronic White Pages telephone directory. The availability in Australia of a single telephone directory in computer format presents a comprehensive and cost-effective listing of residential “land-line” numbers [2, 41]. So as to be representative of the Western Australian population, (1) the survey research centre was given a sample target for location (30 % country) and sex (50 % female), and (2) once contacted, the adult in the household who would next be celebrating a birthday was invited to participate in the study.

The telephone survey was developed by the research team and guided by an online survey of 280 international experts in the field of the arts or arts-health regarding the definition of arts engagement for population based research [16]. The resulting telephone survey was reviewed by a panel of ten experts with experience in market research, the arts and/or public health and carried out by trained interviewers using a computer assisted telephone interview system [42]. The survey took 15 min to complete and included questions about mental well-being (dependent variable), arts engagement over the previous 12 months (independent variable), and eleven possible confounding or effect modifying variables to the arts-mental well-being relationship (i.e. sports engagement, religious activities, holidays, general health, and demographics).

### Dependent variable: subjective mental well-being

The dependent variable in this study was subjective mental well-being and measured by asking respondents the 14 items contained in the Warwick-Edinburgh Mental Well-being Scale (WEMWBS), i.e. *I’ve been feeling … optimistic, useful, relaxed, interested in others, good about myself, close to others, confident, loved, cheerful; I’ve had energy to spare; I’ve been … dealing with problems well, thinking clearly, able to make up my own mind and interested in new things* [43]. WEMWBS measures the mental well-being of the general population. The scale includes hedonic (i.e. happiness, life satisfaction) and eudaimonic (i.e. positive relationships, psychological functioning) items which together measure mental well-being [44]. WEMWBS is designed to assess mental well-being itself and not the determinants of mental well-being (i.e. resilience, problem solving, etc.) [43]. WEMWBS was scored by summing responses (i.e. 1 = none of the time to 5 = all of the time) to each of the 14 items. WEMWBS population scores approximate to a normal distribution, with a minimum possible score of 14 and a maximum score of 70 (population average = 51) [43]. The scale has good face validity, test-retest reliability (Cronbach’s α = 0.83) and internal consistency (Cronbach’s α = 0.89) [43]. WEMWBS has been adapted for cross-cultural use in a number of different countries and has been translated into several languages [45]. Permission to utilise WEMWBS was granted by the University of Warwick.

### Independent variable: hours engaged in the arts

**Fig. 1:**
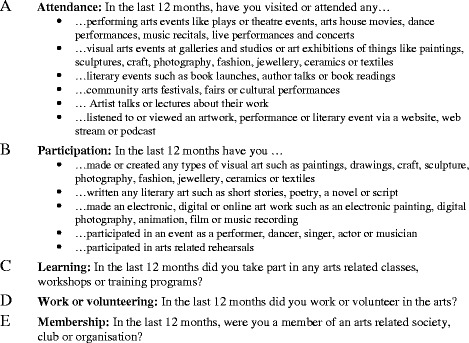
Arts engagement survey questions

The independent variable in this study was total hours engaged in the arts in the last 12 months. Quantifying engagement by asking questions about: (1) activities and events over the last 12 months, and (2) measurement in terms of time, are common in the literature [46–49]. As shown in Fig. 1, arts engagement was measured by asking 14 questions, that is, attendance at arts events (6 questions), participation in the arts (5 questions), learning (1 question), work/volunteering (1 question) and arts related membership (1 question).

For each survey item, respondents were asked if they had engaged in the arts in the previous 12 months (yes/no). If ‘yes’, they were asked to describe the activity or event. Respondents were then asked approximately how many days in the last 12 months they had engaged in each type of activity or event, followed by (on a typical day), how many hours they spent engaging in that activity or event.

### Confounding and effect modifying variables

To control for the influence of confounding and effect modification, information about eleven possible covariates to the arts-mental health relationship were collected (Table 1). This included demographics (i.e. sex, age group, location, income, education, marital status, children) and a self-assessment of general health. Respondents were also asked if during the last 12 months, (1) they partook in a holiday or break from work for two or more consecutive weeks; (2) if at least once a week for most weeks, they attended a religious service/event at a place of worship (e.g. church, mosque, temple); or (3) if at least once a week for most weeks they engaged in sport (i.e. participation in sports activities, and/or attendance at sports events as a spectator, and/or membership of a sports organisation, society or club).

**Table 1:** Respondent demographic, engagement and WEMWBS characteristics (*n* = 702)

Variable	Level	n	%	2011 Western Australian population % (n = 2.3 million people)	Arts engagement prevalence		WEMWBS		
					% Engaged in the arts	*p*-value	Mean score	SD	*p*-value
*Demographics*									
Sex	Male	351	50.0	50 %	75.5	*p* < 0.001	53.2	7.5	NS (*p* = 0.41)
	Female	351	50.0	50 %	90.3		53.6	7.3	
Age Group	18–29 years	63	9.0	15 %	90.5	*p* = 0.013	52.8	6.8	*p* = 0.021
	30–39 years	61	8.7	14 %	85.2		51.5	6.2	
	40–49 years	115	16.4	15 %	89.6		52.6	7.3	
	50–59 years	170	24.2	13 %	84.1		53.1	7.4	
	60 years and over	293	41.7	18 %	77.5		54.4	7.6	
Location	Metropolitan	487	69.4	76 %	83.0	NS (*p* = 0.96)	53.3	7.2	NS (*p* = 0.57)
	Country	215	30.6	24 %	82.8		53.6	7.8	
Household Income ($AUD)	Less than $39,999	195	27.8	26 %	77.4	NS (*p* = 0.10)	53.2	8.6	NS (*p* = 0.87)
	$40,000 to $79,999	123	17.5	21 %	82.1		53.2	7.5	
	$80,000 to $119,999	125	17.8	19 %	87.2		53.4	6.2	
	$120,000 or more	150	21.4	22 %	87.3		54.0	6.8	
	Refused	109	15.5	12 %	82.6		53.3	7.1	
Education	High school or less	293	41.7	44 %	76.1	*p* < 0.001	53.5	7.8	NS (*p* = 0.48)
	Trade certificate or diploma	162	23.1	26 %	86.4		52.8	7.4	
	University degree or higher degree	247	35.2	19 %	88.7		53.7	6.8	
	Refused	0	0.0	11 %					
Married or de-facto relationship	Yes	464	66.1	60 %	84.3	NS (*p* = 0.18)	54.0	6.7	*p* = 0.004
	No	238	33.9	40 %	80.3		52.3	8.5	
Children in household	Yes	202	28.8	59 %	87.1	NS (*p* = 0.59)	53.5	6.6	NS (*p* = 0.55)
	No	500	71.2	41 %	81.2		53.1	7.7	
*In the previous 12 months*									
Sports engagement	Yes	465	66.2	67 %	87.1	*p* < 0.001	53.8	7.0	NS (*p* = 0.06)
	No	237	33.8	33 %	74.7		52.7	8.0	
Attend religious services or events	Yes	267	38.0	-	88.0	*p* = 0.005	53.8	7.5	NS (*p* = 0.30)
	No	435	62.0	-	79.8		53.2	7.3	
Holiday	Yes	384	54.7	-	88.8	*p* < 0.001	54.5	7.1	*p* < 0.001
	No	318	45.3	-	75.8		52.0	7.5	
General health	Very bad	9	1.3	4 %	77.8	NS (*p* = 0.66)	45.9	9.7	*p* < 0.001
	Bad	23	3.3		73.9		45.1	10.0	
	Fair	123	17.5	10 %	80.5		50.5	7.5	
	Good	304	43.3	30 %	84.2		53.4	6.6	
	Very Good	243	34.6	56 %	83.5		56.0	6.5	

*SD* standard deviation, *NS* not statistically significant

- Information not available

### Data analysis

The analysis strategy involved a descriptive investigation of the data followed by Pearson chi-square tests to explore differences by arts engagement and ANOVAs to explore differences in average WEMWBS scores. Arts ‘attendance’ in the previous 12 months was calculated based on respondents indicating they had attended one or more of the six survey items relating to attendance. Similarly, ‘participation’ in the previous 12 months was calculated based on respondents indicating they had participated in one or more of the five survey items relating to participation. A respondent was considered to be engaged in the arts in the previous 12 months (prevalence) if they had attended an arts event, and/or participated in the arts, and/or took part in arts related learning, and/or worked or volunteered in the arts (on a non-professional basis) and/or had been a member of an arts organization, club or society. ‘Total days engaged in the arts in the previous 12 months’ was calculated by summing together the number of days respondents spent attending, participating, learning, working/volunteering or being a member of an arts organization, club or society. ‘Hours per day engaged in the arts in the previous 12 months’, was calculated by first multiplying hours on a typical day by number of days engaged in each type of arts activity over the last 12 months, this was then summed and the total divided by the sum of days engaged in each type of arts activity. ‘Hours per year engaged in the arts’, was calculated by first multiplying hours on a typical day by number of days engaged in each type of arts activity in the previous 12 months and summing each sub-total together. As the distribution of arts engagement was positively skewed (i.e. 17 % did not engage in the arts at all, median = 23 hours/year and 75^th^ percentile = 100 hours/year) and the relationship between mental well-being and arts engagement was non-linear, ‘hours per year engaged in the arts’ was grouped into four categories: no art = 0 hours/year, low arts engagement = 0.1 to 22.9 hours/year, medium arts engagement = 23 to 99.9 hours/year and high arts engagement = 100 or more hours/year. This was followed by linear regression analyses to investigate the association between arts engagement and WEMWBS scores. Overall, three models were fitted. The first model estimated the direct (unadjusted) effect of arts engagement; the second model estimated the effect of arts engagement after adjustment for demographics (i.e. age, sex, location, income, education, marital status and children); and the third model adjusted for demographics, general health, engagement in sport, religious activities and holidays from work. With the exception of the effect modification (i.e. interaction) analyses, results were assessed at the 0.05 level of significance. Effect modification was assessed at the 0.01 level of significance to reduce the possibility of a finding due to chance. The data were analysed using SPSS for Windows (Version 21) and SAS for Windows (Version 9.3).

### Ethics, consent and permissions

Potential respondents were provided with an explanation of the study and invited to provide consent to participate at the beginning of the telephone interview. The respondents who agreed to take part in the study were assured that their answers were confidential, that they could withdraw from the study at any time and that all questions were voluntary. Respondents were made aware that the information collected would be used for research and publication purposes. Permission to conduct this study was granted by The University of Western Australia Human Research Ethics Committee (RA/4/1/2490).

## Results

Overall, 989 phone numbers were called, of which 281 community members (28 %) declined to participate and 708 (72 %) completed an interview. Six respondents were excluded from the analysis as they were professional artists. This resulted in a sample of 702 community members. A sample size of 702 provides 90 % power to detect a difference of 0.25 standard deviations in average mental well-being (WEMWBS) for higher versus lower levels of arts engagement. Table 1 shows the demographic, engagement and WEMWBS characteristics of respondents. For comparison purposes the 2011 Western Australian population profile was also included [50, 51].

Overall, 50 % of respondents were female, 9 % were 18–29 years, 9 % 30–39 years, 16 % 40–49 years, 24 % 50–59 years and 42 % 60+ years. Approximately two thirds of respondents lived in the metropolitan area, were married or in a de-facto relationship, engaged in sport and had not attended a religious service/event in the last 12 months. Twenty-eight percent of respondents had a household income less than AUD$39,999 while 39 % reported their household income was AUD$80,000 or above. Approximately one third of respondents held a university or higher degree. Most respondents described their general health as good or very good (78 %). Approximately half of all respondents had taken a holiday or break from work (for two or more weeks) in the previous 12 months. Most respondents did not have children in their household (71 %).

### Arts engagement

As shown in Table 2, in the previous 12 months, 78 % of respondents had attended an arts event; 48 % participated in the arts; 11 % took part in arts related learning; 11 % worked or volunteered in the arts and 10 % were a member of an arts society, club or organisation. The highest number of respondents attended a performing arts (63 %) or visual arts event (51 %). On average, in the previous 12 months, respondents spent 16 hrs attending arts events; 63 hrs making or creating art; 5 hrs learning about the arts; 9 hrs working or volunteering in the arts (on a non-professional basis) and 7 hrs as a member of a arts society, club or organisation. The prevalence of arts engagement was 83 %. Respondents spent an average of 101 hours/year engaging in an arts activity or event (SD = 206, median = 23 hrs, minimum = 0 h, maximum = 1572 hrs).

**Table 2:** Prevalence, days and hours engaged in the arts in the previous 12 months (*n* = 702)

Arts engagement measure		Attendance	Participation	Learning	Work or volunteer^a^	Member	Overall arts engagement
Prevalence of arts engagement in the previous 12 months		77.8 %	48.4 %	11.4 %	11.0 %	10.3 %	82.9 %
Days engaged in the arts in the previous 12 months	Mean	5.66	20.67	1.76	2.63	2.35	33.06
	SD	8.33	46.17	7.94	10.82	9.19	56.93
	Median	3.00	0.00	0.00	0.00	0.00	9.00
	IQR	6.00	21.00	0.00	0.00	0.00	37.00
Hours per day engaged in the arts in the previous 12 months	Mean	2.28	1.39	0.39	0.44	0.29	2.44
	SD	1.84	1.94	1.29	1.61	0.95	1.81
	Median	2.00	0.00	0.00	0.00	0.00	2.07
	IQR	2.00	2.00	0.00	0.00	0.00	1.85
Hours engaged in the arts in the previous 12 months	Mean	16.09	63.32	5.32	9.04	7.02	100.80
	SD	25.11	170.99	26.93	45.47	30.92	205.99
	Median	8.00	0.00	0.00	0.00	0.00	23.00
	IQR	18.00	48.00	0.00	0.00	0.00	96.00

*SD* standard deviation, *IQR* interquartile range

^a^Non-professional artist

When the data was grouped, 17 % of respondents did not engage in the arts, 33 % had low arts engagement (0.1 to 22.9 h/year), 24 % had medium arts engagement (23 to 99.9 h/year) and 26 % had high arts engagement (100 or more hours/year). As shown in Table 1, females were significantly more likely to be engaged in the arts than males (*χ* ^2^ = 27.2 df = 1 *p* < 0.001); as were younger compared to older respondents (*χ* ^2^ = 12.7 df = 4 *p* = 0.013). Arts engagement also significantly increased by education (*χ* ^2^ = 16.7 df = 2 *p* < 0.001), and was more likely in those who also engaged in sport (*χ* ^2^ = 17.1 df = 1 *p* < 0.001), attended religious services/events (*χ* ^2^ = 7.9 df = 1 *p* = 0.005) and those who had taken a holiday or break from work for two or more weeks in the previous 12 months (*χ* ^2^ = 20.8 df = 1 *p* < 0.001).

### Arts engagement and mental well-being

The WEMWBS mean score for respondents was 53.4 (median = 54.0, SD = 7.4, minimum = 21.0, maximum = 70.0). As shown in Table 1, respondents who were married or in a defacto relationship had higher average WEMWBS scores than unmarried respondents (F = 8.57 df = 1 *p* = 0.004); as did older compared to younger respondents (F = 2.91 df = 4 *p* = 0.021). Average WEMWBS scores were also significantly higher for those with good general health (F = 24.63 df = 4 *p* < 0.001) and those who had taken a holiday or break from work for two or more weeks in the previous 12 months (F = 20.23 df = 1 *p* < 0.001).

As shown in Table 3, Model 1 (unadjusted), respondents who engaged in a high level of arts engagement had higher average WEMWBS scores (54.8) than those who did not engage in the arts (52.8) or who had low (52.6) or medium (53.4) levels of engagement. With the exception of having “energy to spare”, for 13 out of the 14 WEMWBS questions, item means were highest for high arts engagement respondents compared to other levels of engagement, especially regarding optimism, interest in other people, thinking clearly, feeling loved, being interest in new things and feeling cheerful.

**Table 3:** Association between subjective mental well-being and arts engagement (hours per year)

	Regression models								
	1. WEMWBS (Unadjusted Model)			2. WEMWBS adjusted for demographics (age, sex, location, income, education, married, children)			3. WEMWBS adjusted for demographics, general health, sports engagement, religious activities & holidays		
	β	SE	*p*-value	β	SE	*p*-value	β	SE	*p*-value
None vs High engagement	−2.01	0.87	0.0206	−1.94	0.90	0.0307	−1.60	0.85	0.0612
Low vs High engagement	−2.25	0.73	0.0022	−2.22	0.74	0.0026	−2.57	0.69	0.0002
Medium vs High engagement	−1.45	0.78	0.0650	−1.40	0.79	0.0755	−1.74	0.73	0.0178
Arts engagement	Mean Scores			Mean Scores			Mean Scores		
None/no art (*n* = 120)	52.83			52.87			53.35		
Low engagement (*n* = 230)	52.59			52.59			52.38		
Medium engagement (*n* = 172)	53.40			53.41			53.21		
High engagement (*n* = 180)	54.84			54.81			54.95		
	F value	df	p-value	F value	df	*p*-value	F value	df	*p*-value
Overall Test – 4 groups *(none, low, medium, high arts engagement)*	3.48	3	0.0157	3.25	3	0.0215	4.67	3	0.0031
Overall Test – 3 groups *(none, low, medium arts engagement)*	0.59	2	0.5548	0.60	2	0.5482	1.03	2	0.3582
Overall Test – 2 groups (not high versus high arts engagement)^a^	9.26	1	0.0024	8.50	1	0.0037	11.74	1	0.0006

^a^High arts engagement = 100 or more hours/year; Not high engagement (none, low, medium) = 99.99 or less hours/year

As shown in Table 3, Model 3, after adjustment for demographics, general health, engagement in sport, religious activities and holidays (Overall Test - 4 groups *p* = 0.003), it was found that people with a high level of arts engagement (i.e. 100 or more hours/year) had higher WEMWBS scores than those with none, low or medium engagement. In comparison, people with none, low and medium levels of arts engagement were found to have similar WEMWBS scores (Model 3, Overall Test - 3 groups *p* = 0.358), therefore a trend analysis was not applicable. Overall, the relationship between arts engagement and WEMWBS was nonlinear with evidence of a minimum threshold at 100 or more hours of arts engagement per year (i.e. high engagement vs non/low/medium engagement, Model 3, Overall Test - 2 groups *p* = 0.0006). A small decrease in significance level was found after adjustment for confounding variables, while effect modification by demographic, sport, religious activites, holidays or general health was not found to be significant. In general, respondents who participated in 100 or more hours of arts engagement per year had WEMWBS scores approximately two points higher than other levels of engagements (i.e. one third of a standard deviation).

## Discussion

This is the first study to quantify the dose–response relationship between recreational arts engagement and mental health in a general population and is a starting point as to whether a population-based arts engagement strategy can be utilised to improve the mental well-being of the general population. Encouraging the population to “shift” their behaviour to encourage well-being is a common strategy in the public health literature as this approach can benefit more individuals (overall) than targeting only high risk individuals. For example, a population approach to reducing overweight/obesity might be to encourage the general population to modify their diet and engage in adequate levels of physical activity. Likewise, population-based strategies to improving mental health could be to encourage the general population to engage in activities and behaviours (e.g. via the arts) that foster well-being.

The literature suggests that two hours per week of volunteering can increase mental well-being in older adults [52]. A dose–response effect between psychosocial well-being and leisure related physical activity (i.e. at least two hours/week) has also been proposed [12]. After controlling for a variety of covariates, an association between subjective mental well-being (WEMWBS) and arts engagement was found in this study. The relationship was nonlinear with evidence of a minimum time threshold at 100 or more hours of arts engagement per year (i.e. two or more hours/week). The possibility of a threshold level of arts engagement to obtain health benefits was alluded to by McCarthy and Ondaatje who suggested that emotional gains were likely to accrue once an individual attained a certain level of understanding and knowledge about an art-form, activity or event [53]. The group of respondents who participated in 100 or more hours of arts engagement per year (high arts engagement) had an average WEMWBS score approximately two points higher than other groups highlighting the possibility of a dose–response health effect.

This paper provides three important findings for health professionals, clinicians, researchers, policy makers and the general population, and as Western Australia is representative of the broader Australian population, these findings could be generalised to other Australian states/territories and to countries with similar mental-health and socio-demographic profiles to Australia. First, 100 or more hours/year (i.e. two or more hours/week) of arts engagement may have the potential to enhance mental well-being in the general population. Second, when engaging in creative activities and events, the amount of time engaged in the arts, or ‘the art dose’, may be important in obtaining mental health benefits. Third, in time, if the relationship between hours engaged in the arts and good mental health is found to be causal, there is potential for new and innovative ‘time based’ arts-mental health campaigns, such as those used to promote the health benefits of physical activity. For example, in Western Australia, the National Heart Foundation and the Department of Health were successful at developing and implementing the ‘Find 30 – it’s not a big exercise’ and the ‘Find 30 everyday’ campaigns to promote the health benefits of physical activity to the general population and increase awareness of the amount of moderate-intensity physical activity needed for good health (i.e. 30 min each day) [54].

## Conclusion

Strengths of this population-based study include its large sample size, high response, detailed quantification of arts engagement (hours per year), adjustment for a wide range of confounders/effect modifiers to the arts-mental health relationship and that the sample was representative of the Western Australian population in terms of sex, location, income, marriage and sports engagement. Limitations of this study were that only people who had phone numbers listed in the Australian Electronic White pages were asked to participate and that there was an over representation of older adults, people with a university or higher degree and people without children in their household. As this study was observational, it also precludes our ability to determine causality. For researchers to determine whether or not a causal relationship exists between arts engagement and mental well-being the following needs to occur: (1) a plausible conceptual framework of the relationship between arts engagement and mental health including possible confounders and effect modifiers needs to developed; (2) good study designs, that consider coherence and temporal order, need to be employed; and (3) the strength, consistency and specificity of the arts-mental health relationship needs to be considered [55]. The cross-sectional design of this study was cost-effective and adequate as a starting point for assessing the relationship between arts engagement and mental well-being however, future research should extend on current findings via more robust study designs (e.g. prospective cohort studies). Enablers and barriers to the arts-mental health relationship should also be investigated, as should the influence of art form (e.g. visual arts, performing arts, etc.), type (i.e. active versus receptive engagement), and mode of engagement (e.g. attendance, participation, etc.) to determine which elements have the most impact on mental well-being. As arts engagement has also been linked with social and physical health benefits [31], further research is needed to quantify and explore the arts-social health and the arts-physical health relationship. The ability of the arts to promote, maintain and improve population health requires further investigation as arts activities and events have the potential to contribute to health promotion strategies and have implications for innovative public health policy and practice.

## Abbreviations

Hours/week: hours per week
Hours/year: hours per year
IQR: interquartile range
NS: not statistically significant
SD: standard deviation
WEMWBS: Warwick-Edinburgh Mental Well-being Scale
